# New insights into the degradation mechanism of metal-organic frameworks drug carriers

**DOI:** 10.1038/s41598-017-13323-1

**Published:** 2017-10-13

**Authors:** X. Li, L. Lachmanski, S. Safi, S. Sene, C. Serre, J. M. Grenèche, J. Zhang, R. Gref

**Affiliations:** 1 Institut des Sciences Moléculaires d’Orsay, UMR 8214 CNRS, Université Paris-Sud, Université Paris-Saclay, Orsay, 91405 France; 2 Malvern Instruments, 30 rue Jean Rostand, Orsay, 91405 France; 3 Institut de Matériaux Poreux de Paris, FRE 2000 CNRS ENS-ESPCI, PSL Research University, Paris, 75005 France; 4 Institut des Molécules et des Matériaux du Mans (IMMM) - UMR 6283 CNRS, Le Mans Université, Le Mans, 72085 France; 5 Shanghai Institute of Materia Medica, Chinese Academy of Sciences, Shanghai, 201203 China

## Abstract

A versatile method based on Raman microscopy was developed to follow the degradation of iron carboxylate Metal Organic Framework (MOF) nano- or micro-particles in simulated body fluid (phosphate buffer). The analysis of both the morphology and chemical composition of individual particles, including observation at different regions on the same particle, evidenced the formation of a sharp erosion front during particle degradation. Interestingly, this front separated an intact non eroded crystalline core from an amorphous shell made of an inorganic network. According to Mössbauer spectrometry investigations, the shell consists essentially of iron phosphates. Noteworthy, neither drug loading nor surface modification affected the integrity of the tridimensional MOF network. These findings could be of interest in the further development of next generations of MOF drug carriers.

## Introduction

Metal-organic frameworks (MOFs) are a recent class of ordered porous materials formed by the coordination of metal clusters and organic ligands. Due to their versatile chemistry, high pore volumes and large surface areas, MOFs attracted growing interest in various areas, including gas storage^1,2^, catalysis^3–5^, separation science^6^, sensing^7,8^, ionic conduction^9^ and more recently, biomedicine^10–13^. Lately, biodegradable micro- or meso-porous iron polycarboxylate MOFs were employed as drug carriers^10,11,14–18^. More particularly, iron trimesate MIL-100 and iron terephthalate MIL-101 (MIL stands for Materials of Institute Lavoisier) were considered among the most efficient materials to load drugs because of their large pore sizes (24–29 and 27–34 Å for MIL-100 and MIL-101, respectively)^19^. As an example, ibuprofen payloads reached up to 35 wt% and 140 wt% for MIL-100 and MIL-101, respectively^11^. However, nano-scaled MOFs (nanoMOFs) made of biocompatible MIL-101(Fe) suffer from a very fast degradation in phosphate buffer saline (PBS) hampering their applications for controlled drug delivery^20^. In contrast, MIL-100(Fe) nanoMOFs degraded in a more progressive manner compatible with biomedical applications and were well tolerated *in vivo* ^21^. A variety of drugs have been successfully incorporated into MIL-100(Fe) nanoMOFs, including antiviral drugs (cidofovir^10^, azidothimidine triphosphate (AZT-TP) and azidothimidine monophosphate (AZT-MP))^15,22^, anticancer drugs (busulfan^16^, topotecan^18^, doxorubicin^23^ and gemcitabine-monophosphate (Gem-MP)^24^), biological gases (nitric oxide)^25^, caffeine^26^, aspirin^27^ and metallodrugs^28^.

The highest loading efficiencies were reported in the case of phosphated drugs, including AZT-TP, AZT-MP and Gem-MP^15,22,24^. MIL-100 were able to soak the drugs within minutes from their aqueous solution, reaching elevated drug loadings (around 30, 25 and 36 wt% for Gem-MP, AZT-TP and AZT-MP, respectively) and encapsulation efficiencies higher than 98%. It was demonstrated that coordination of the drug’s phosphate groups on the iron(III) Lewis acid sites in MIL-100 nanoMOFs played a main role, leading to an efficient encapsulation^22,24^. Indeed, the same drugs without phosphate groups were poorly incorporated^22^.

Phosphate molecules are essential components in every living organism. When non-controlled, strong interactions between phosphates and MOFs can lead to a fast degradation and thus to “burst” drug release. Therefore, it is of outmost importance in the development of MOFs drug carriers to gain deep understanding on their degradation in the presence of phosphates. It was shown that MIL-100(Fe) particles exhibit good colloidal stability and biodegradability in a series of simulated physiological fluids^29^ and the degradation of nanoMOFs has been studied in different cell culture medium as reported^29^. There was around 10 wt% of trimesate released in Dulbecco’s Modified Eagle’s medium (DMEM), but only around 5% released in Minimum Essential Medium Eagle (MEM). Trimesate release was dependent on the composition of the cell culture medium, especially on its content in phosphates. Intravenously administered nanoMOFs in rats were rapidly sequestered by the liver and spleen, then further biodegraded and directly eliminated in urine or feces^30,31^. The trimesate linkers were progressively eliminated in urine^21^. All these studies have shown the biodegradability of nanoMOFs for potential biomedical applications, but their degradation mechanism is not fully understood yet. Recently, Bezverkhyy *et al*.^32^ demonstrated that MIL-100(Fe) nanoMOFs maintained their crystalline structures while heated in water at 100 °C for 48 h, but partially degraded. On the contrary, at neutral pH the particles degraded within one hour yielding poorly crystallized iron oxide (ferrihydrite). These experiments were however conducted in media without phosphate groups, not mimicking the biological conditions. In other studies, it was shown that the MIL-100(Fe) nanoMOFs progressively disintegrated in PBS because of the strong interactions between phosphates and iron and that this degradation was well correlated to drug release^15,18,23^.

One can also tailor the MOF particle size according to the targeted application and route of administration even leading to micron-sized MOFs (microMOFs) particles^33^. NanoMOFs had been reported to exhibit a narrower particle size distributions than microMOFs but were more prone to faster degradation because of larger surface areas^33^. However, no study dealt yet with the influence of the MOF particle size on the degradation mechanism. Moreover, the investigations on MIL-100(Fe) MOFs degradation were conducted so far using batches of particles, but never on individual particles. In addition it has not been assessed yet at the particle level if drug loading or surface modification impacts the stability of the nanoMOF. It is therefore of main interest to study the degradation of individual MOFs.

To address this goal we have used here Raman microscopy to monitor *in situ* the degradation in phosphate-containing media of individual MIL-100(Fe) MOF particles of different sizes. This versatile method combines the advantages of both Raman spectroscopy and optical microscopy. The utility of Raman microscopy as a rapid, label-free, and non-destructive method was proven in many applications where chemical analysis and imaging are simultaneously required. This technique allows to qualitatively or quantitatively characterize the chemical composition and structure of a sample. In the pharmaceutical field, Raman microscopy is used mainly to characterize the distribution of molecules of interest in tablets, to determine the crystallinity of the materials, to investigate purity and chemical composition of complex devices.

In this study, MOF batches were analyzed in terms of particle size distribution and shape. Raman spectra of individual particles were recorded, enabling to assess the homogeneity of the MOF population in terms of chemical composition and morphology. Of note, the degradation of each particle in PBS was monitored up to 8 days, enabling to detect eroded and intact zones in the same particle. Finally, we showed that the integrity of the MIL-100(Fe) MOFs was maintained after drug loading and surface modification.

## Results and Discussion

### Synthesis and characterization of nanoMOFs

**Figure 1:**
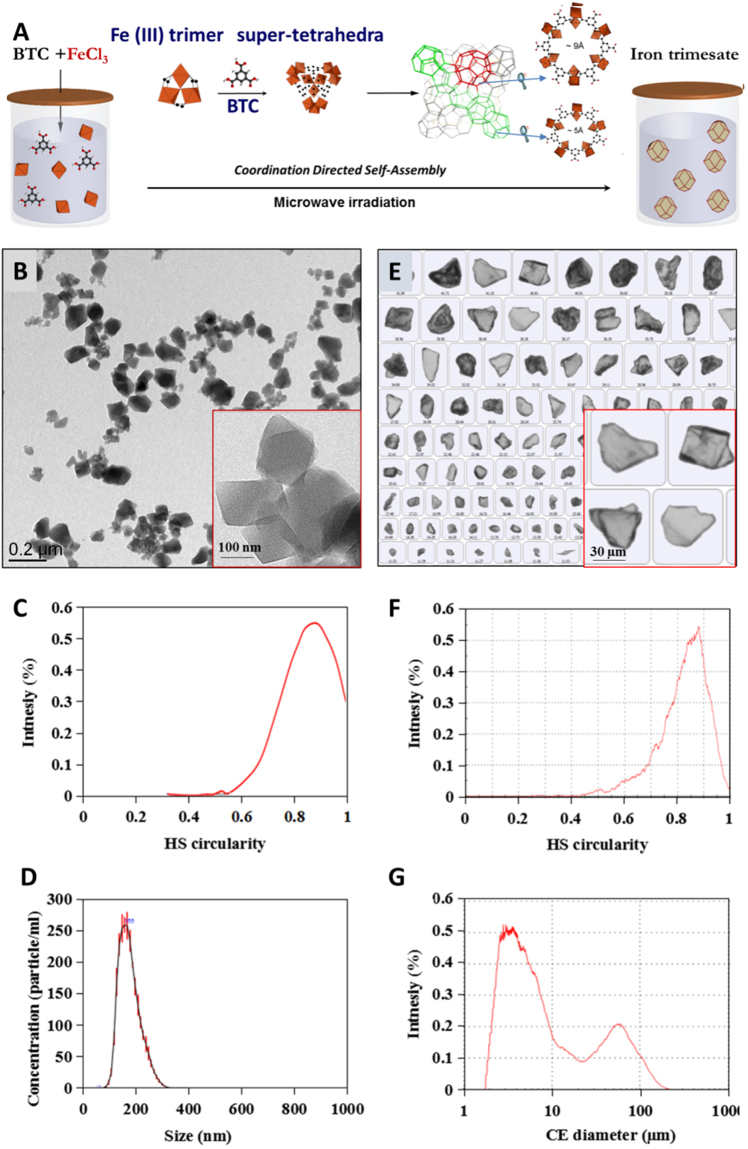
Schematic representation of the “green” hydrothermal synthesis of MOFs (**A**) and their morphological characterization, including TEM images of nanoMOFs (**B**) and optical images of microMOFs (**E**); circularity for nanoMOFs (**C**) and microMOFs (**F**) calculated from more than 8000 particles for nanoMOFs and 2000 particles for microMOFs, respectively, as well as size distributions for nanoMOFs (**D**) and microMOFs (**G**). Iron polyhedra, oxygen, carbon atoms are in orange, red and black, respectively.

Nano-scaled MIL-100(Fe) particles were successfully prepared as reported by a solvent-free and fluoride-free “green” hydrothermal microwave assisted method using as reactants iron chloride and trimesic acid^34^. As schematized in Fig. 1A, the spontaneous coordination of Fe(III) trimers and trimesic acid generates hybrid supertetrahedra which further assemble into a zeotypic architecture consisting of: i) small mesoporous cages (Ø = 25 Å), delimited by pentagonal openings (5.6 Å) and ii) large mesoporous cages (Ø = 29 Å) accessible through both pentagonal and larger hexagonal windows (8.6 Å)^11^. The nanoMOFs’ Powder X-Ray Diffraction (PXRD) patterns and nitrogen porosimetry BET surface areas (S_BET_ = 1690 ± 50 m^2^/g) are in agreement with previously published data^15,19^.

As illustrated in Fig. 1B, the nanoMOFs appear as small crystals with a faceted morphology and rhombic structures. The Transmission Electron Microscopy (TEM) analysis of more than 8000 nanoMOFs particles allowed estimating their average diameter of 187 ± 55 nm and a mean circularity of around 0.89 (Fig. 1C) in agreement with the sharp-edged shapes observed by TEM (Fig. 1B). The mean diameter of the nanoMOFs measured by dynamic Light Scattering (DLS, number values) is 216 ± 21 nm with a polydispersity index (PdI) of 0.131.

In a complementary approach, the size of individual nanoMOFs was determined by nanoparticle tracking analysis (NTA), a method which combines an optical microscope and a laser illumination unit^35^. The mean diameter of each particle was calculated from its trajectory in Brownian motion, using the Stokes–Einstein equation. The obtained mean diameter of 196 ± 59 nm (Fig. 1D) was in agreement with those obtained by both DLS and TEM studies. These findings support the fact that the nanoMOFs have a relatively narrow particle size distribution.

### Synthesis and characterization of MIL-100(Fe) microMOFs

MicroMOFs were synthesized by a similar method as nanoMOFs, but with longer reaction times to allow the crystals to grow larger. The microMOFs displayed a crystalline structure according to PXRD patterns and nitrogen porosimetry BET surfaces of around 1700 ± 60 m^2^/g. More than 2000 individual particles were observed by Raman microscopy. Typically, the microMOFs appeared as crystals with irregular shapes (Fig. 1E). Statistical analysis showed that microMOFs possess a mean circularity (0.84) slightly lower than the nanoMOFs (Fig. 1F). MicroMOF samples contained: i) a main population with an average diameter of around 3 µm and ii) a smaller population of much larger particles of size around 60 µm (Fig. 1G). Contrary to nanoMOFs which had a narrow size distribution, microMOFs possess thus a bimodal size distribution, possibly because of the difficulty to control crystal nucleation and growth, a process dependent on many experimental parameters. These findings highlight the importance to perform individual particle analysis to gain insights into the degradation of MIL-100(Fe) particles of different sizes.

### Degradation of nanoMOFs

To study the effect of phosphates on nanoMOF degradation, the crystalline particles were incubated in PBS with different phosphate concentrations and in water, as a control. NanoMOFs incubated in PBS (1.19 mM) at 37 °C experienced a rapid degradation, releasing large amounts (34 ± 3 wt%) of their constituting ligand, trimesate, after only 6 h. These data are in agreement with previously reported studies, where 29.9 ± 2.1 wt% trimesate was released under similar conditions^28^. Comparable amounts of ligands (31 ± 3 wt%) were released but after two days of incubation in PBS when diluted ten times (0.12 mM)(for details see SI section). In both cases, the concentration of nanoMOFs was 0.5 mg/mL, corresponding to 0.1 mM Fe. This highlights the influence of the concentration of phosphates on the degradation mechanism: the higher the concentration of phosphates, the faster the degradation of nanoMOFs in PBS.

In contrast, nanoMOFs released negligible amounts (less than 2 wt%) of their trimesate constituting ligand within two days incubation in pure water at 37 °C, in agreement with previous investigations^15,22^, where less than 1 wt% trimesate was released after incubation for three days. This is in accordance with the nanoMOFs’ diameter that remained practically unchanged over two days incubation in water (224 ± 25 nm).

Note that nanoMOFs lost their crystallinity in PBS within only 6 h, whereas they maintained an intact crystallinity in water, as indicated by PXRD results (Fig. S1).

**Figure 2:**
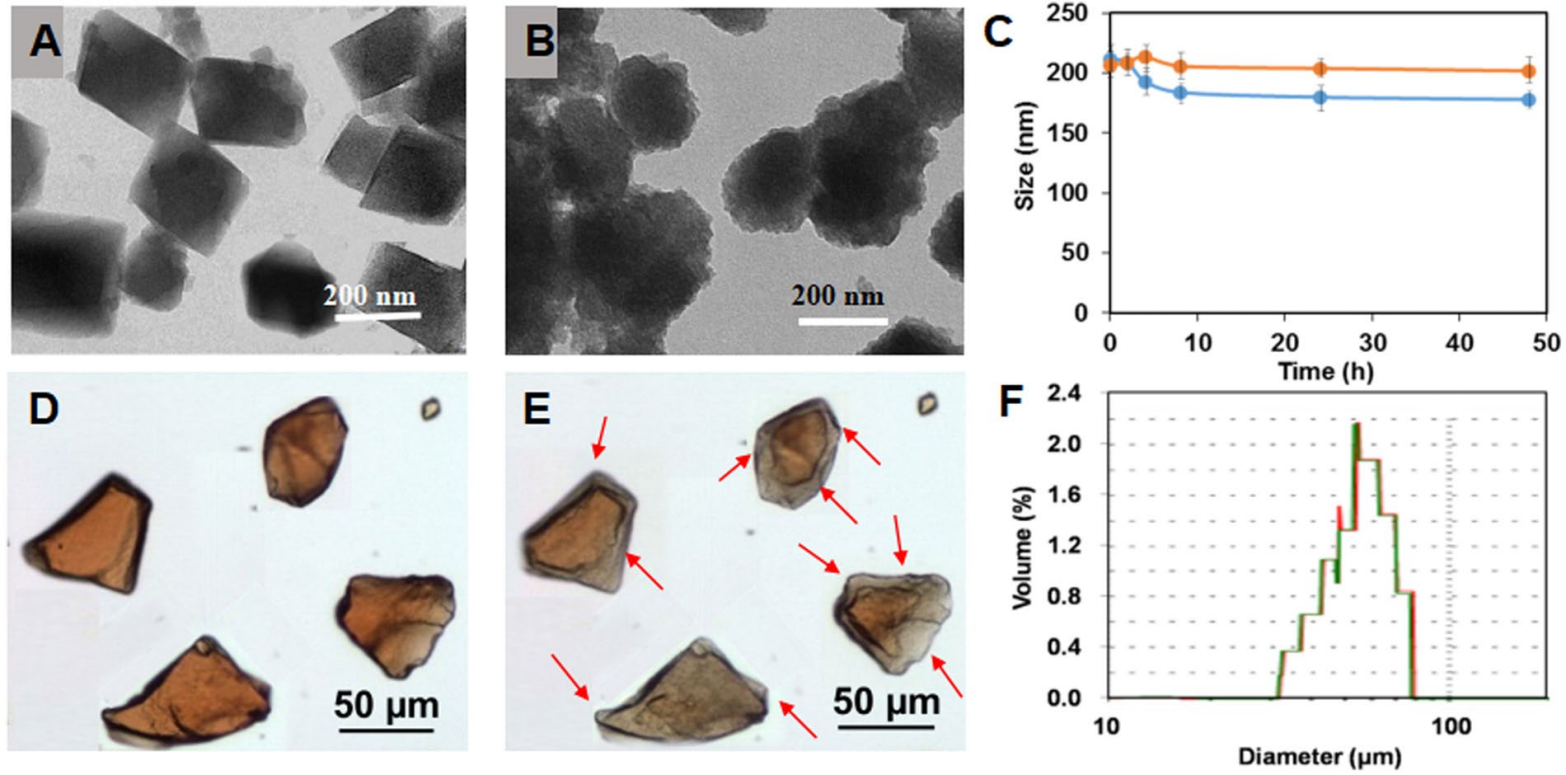
Morphology of MIL-100(Fe) nano-and microMOFs and size variations after incubation in water or PBS. TEM images of nanoMOFs before (**A**) and after (**B**) degradation in PBS (1.19 mM) for 6 h. Size variation (**C**) of nanoMOFs (100 µg/mL) in MilliQ water (orange) and PBS (0.12 mM, blue) for 2 days. Images of microMOFs observed by Raman-microscopy before (**D**) and after (**E**) degradation in PBS (11.9 mM) for 8 days; size distribution (**F**) of microMOFs before (green) and after (red) degradation.

These observations were well corroborated by TEM investigations, evidencing the nanoMOF morphological aspects before (Fig. 2A) and after (Fig. 2B) degradation in PBS. Noteworthy, degradation in PBS leads to a progressive rounding (Fig. 2B) of the nanoMOFs sharp initial edges (Fig. 2A), in agreement with a progressive disorganization of the crystalline 3D structure. Interestingly, DLS measurements showed that despite the degradation, the nanoMOFs maintained practically the same mean diameter (Fig. 2C), with a moderate decrease in size from 221 ± 24 nm to 187 ± 22 nm after two days incubation in PBS, despite the fact more than 30% of their constitutive ligand has left the particles, as shown above.

In a nutshell, these studies confirm that these iron carboxylate based nanoMOFs are stable in water, but degrade in PBS with a progressive departure of the constitutive organic linkers associated with an amorphization. However, it is remarkable that despite their dramatic morphological and structural changes in PBS, nanoMOFs maintain practically unchanged diameters over two days. Based on these findings, it was interesting to further investigate the degradation of larger particles (microMOFs) in PBS.

### Degradation of MIL-100(Fe) microMOFs

As shown in Fig. 2D, MIL-100(Fe) microMOFs are red-orange crystals with irregular shapes. The red color is attributed to Fe^3+^ species in the [Fe_3_O(OH)(H_2_O)_2_]^6+^ clusters linked by carboxylate anions^27^. Contrary to nanoMOFs, microMOFs degrade very slowly and therefore concentrated PBS (11.9 mM) was used to accelerate the process. After 8 days of incubation in this medium, the particles changed color (Fig. 2E). However, surprisingly, the size of the particles monitored by Raman microscopy remained unchanged (less than 0.5% variation in all dimensions) (Fig. 2F) as it was previously shown in the case of nanoMOFs. Grey regions (arrows in Fig. 2E) were clearly observed, whereas the core of the particles maintained its initial red color.

In a nutshell, MOF particles degraded with the formation of a grey shell delimiting a red-colored core. Raman microscopy was further used to study the composition of these different regions in the same particle.

### Individual MIL-100(Fe) particle degradation

**Figure 3:**
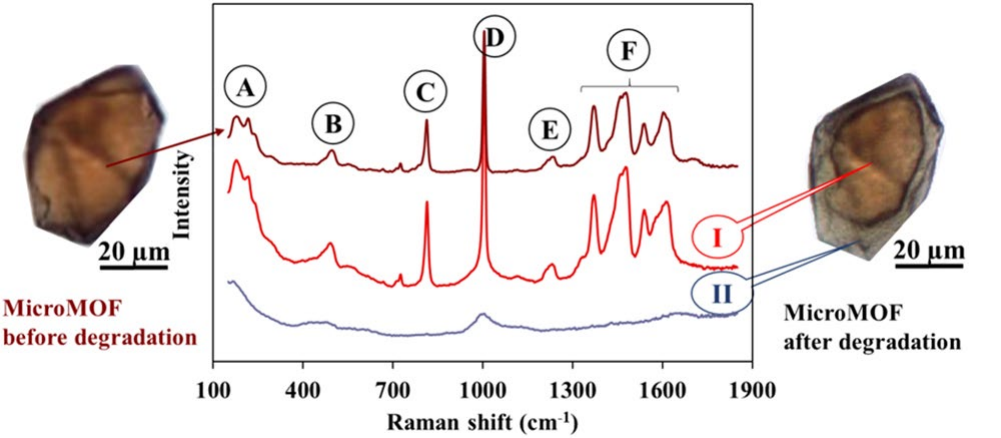
Raman spectra of a single microMOF particle of around 50 microns before (brown) and after (red and blue) degradation for 8 days in PBS (11.9 mM). Raman spectra were recorded on the same particle, both in the red core (region I) and in the degraded grey shell (region II).

Typical Raman spectra of microMOFs show the specific MIL-100(Fe) peaks in the 100–1900 cm^−1^ region (Fig. 3). The peak A at 210 cm^−1^ is attributed to the ordered crystalline iron-based structure, whereas the B bands from 450 cm^−1^ to 600 cm^−1^ are related to lattice vibrations and network binding modes. The principal peaks in Fig. 3 correspond to the fingerprints of the trimesate linker, the aromatic ring peaks at 800 cm^−1^ (peak C) and 1000 cm^−1^ (peak D). The E peaks around 1200 cm^−1^ can be assigned to the C–O–Fe stretching of Fe-trimesate, whereas F bands ranging from 1400 cm^−1^ to 1600 cm^−1^ are related to the H–O–H bonding vibrations, which indicate that the Fe-trimesate network contains coordinated water.

Figure 3 presents a typical image of a microMOF particle before (left) and after 8 days degradation in PBS (right). The morphology was dramatically changed with the appearance of a sharp transition delimiting a red-colored core (region I) from a grey shell (region II). Of note, Raman microscopy enabled monitoring Raman spectra in both I and II regions. The spectrum of region I consisted of peaks very similar to those characteristic of non-degraded MOFs, with the exception of peak D which was broadened at its base. On the contrary, in region II, all peaks disappeared and only a large peak D could be detected. The disappearance of band A indicates an amorphous phase formation. Peaks B shifted to lower wavelengths, become much broader, and less resolved, suggesting a change in coordination environment. The disappearance of the well resolved peaks C could be interpreted as a proof of trimesate release. The band D which was broadened but did not disappear could be ascribed to symmetric and asymmetric stretching vibrations of Fe–O–P bonds, strongly indicating the formation of phosphate complexes by coordination with phosphate ions^32^. This is also in agreement with the disappearance of peak F in the eroded region II, indicating that coordinated water faded away.

In conclusion, Raman spectra gave useful information regarding MOFs degradation in PBS, suggesting that the most probable degradation mechanism is the competitive replacement of trimesate by phosphate ions in PBS solution. Advantageously, Raman microscopy enabled obtaining spectra in different regions on the same particle. To gain further insights on the degradation mechanism, single particles were tracked over their degradation in PBS during 8 days.

**Figure 4:**
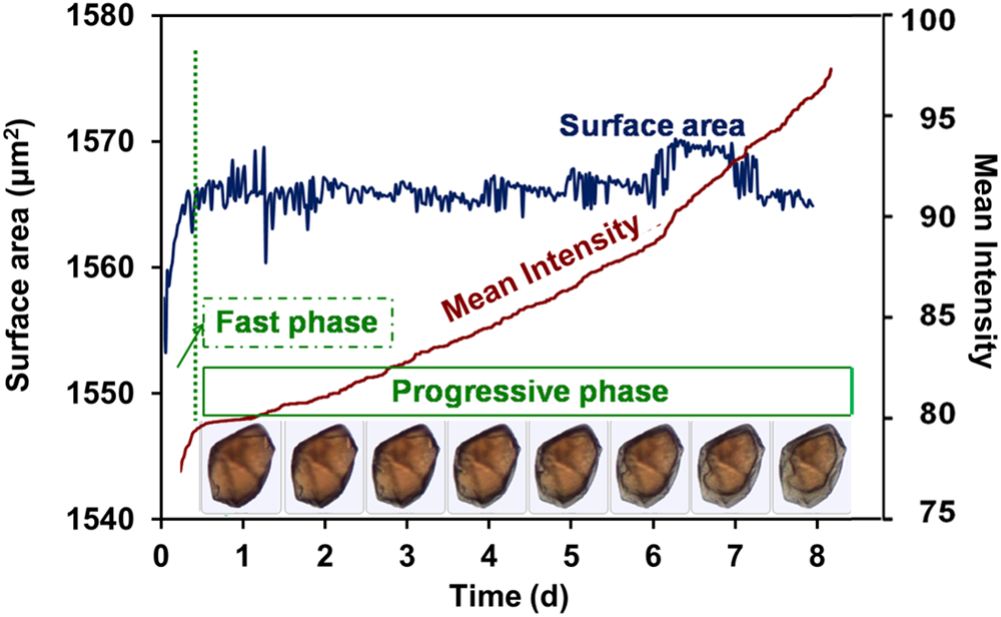
Kinetic study of the degradation of a microMOFs (size around 50 µm in length) in PBS (11.9 mM) at room temperature. Particle surface area and mean intensity were recorded each 30 min up to 8 days. A fast degradation phase was observed in the first 6 h with a rapid increase in mean intensity. A second phase occurred in the following 8 days with the formation of an eroded zone with a clear color change (progressive phase).

Automated imaging was used to gain a deeper understanding of the kinetics of the degradation process of each selected particle, since it can provide a direct measurement of the size, shape, color, surface area, and circularity of each particle. Images were captured every half an hour during 8 days to track the degradation process. Selected images illustrate the typical morphologies of one single particle during degradation (Fig. 4). Although shells (eroded zones) with a grey color were clearly formed and progressively penetrated into the particles, no significant variation in size could be detected.

Additionally, the variation of mean intensity and surface area was also investigated. Mean intensity is a precise parameter to evaluate the color of a particle, based on the amount of light reflected by a particle. The darker the particle, the less is the reflected light, resulting in lower intensities. Figure 4 shows a sharp increase in the intensity of a MOF particle in the first 6 h, indicating a rapid color change, which was too subtle to be observed by the naked eye. This fast phase was followed by a slower increase in mean intensity, accompanied by no detectable surface area variation (Fig. 4, progressive phase).

It can be speculated that in the first hours after incubation, a fast coordination reaction occurred between phosphates in the incubation medium and accessible iron sites at the MOFs surface. This phase was followed by a slower diffusion of phosphates in the eroded region. The advancement of the degradation front was presumably limited by the diffusion of the reacting species (phosphates) from the media into the MOFs and by the diffusion of released trimesate out of the matrix.

### Mössbauer spectrometry

**Figure 5:**
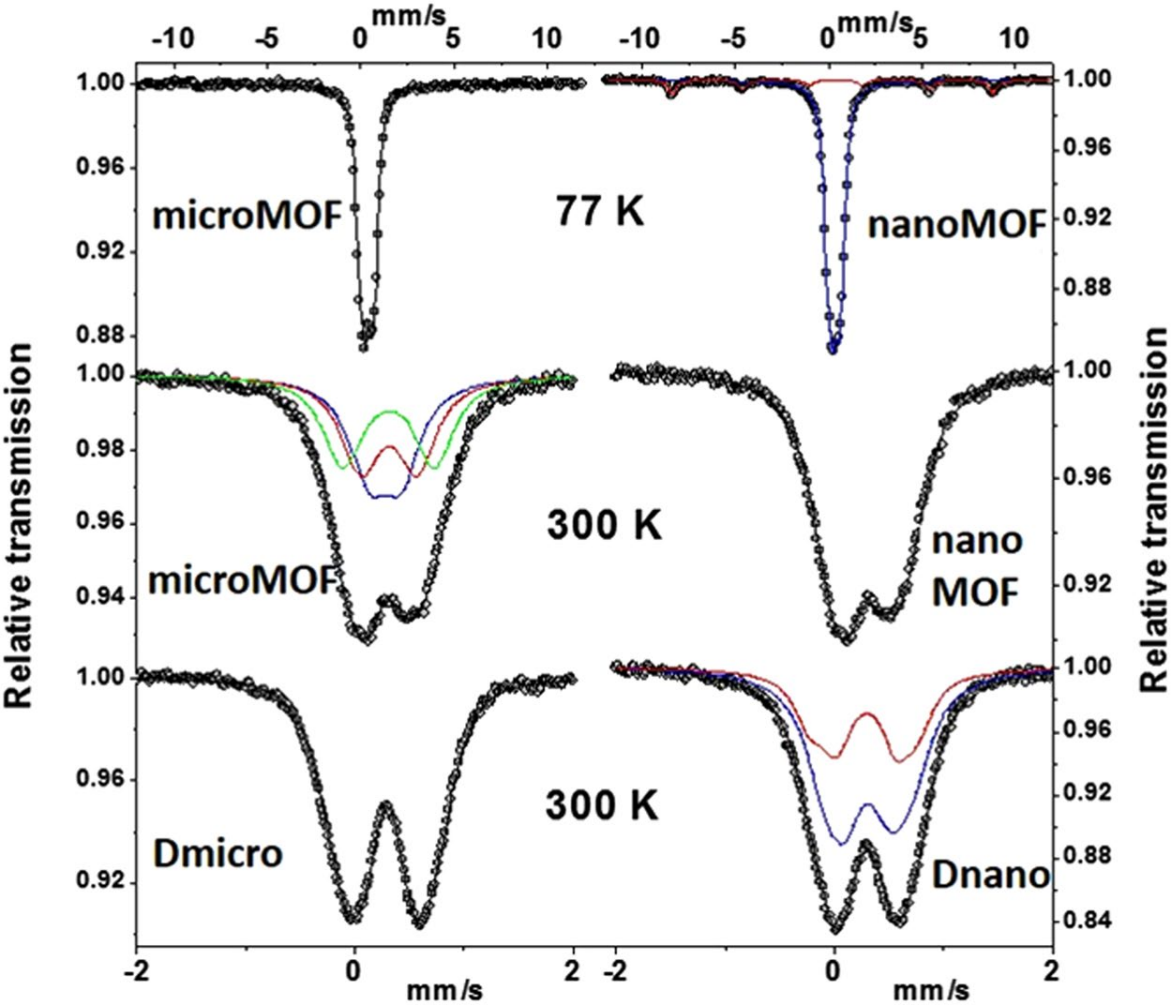
Mössbauer spectra of nano-and microMOFs obtained at 77 K and at 300 K. Spectra of degraded nanoMOFs (Dnano) and microMOFs (Dmicro) obtained at 300 K.

^57^Fe Mössbauer spectrometry was applied to MIL-100(Fe) particles to probe several chemical features of interest, such as the Fe oxidation state, the type of Fe species, the possible presence of Fe-containing phases different from of MIL-100(Fe) and any Fe oxide resulting from degradation. Mössbauer spectra were recorded at 300 and 77 K with different velocities and only typical Mössbauer ones are illustrated in Fig. 5. The hyperfine structures result essentially from the presence of quadrupolar components. The refined values of the isomer shift indicate unambiguously the presence of only high supersaturation (HS) Fe^3+^ species whatever the sample (nano-and microMOFs) and their degradation state. It could be concluded that no Fe reduction phenomenon occurred in PBS.

At 300 K, the hyperfine structures of the nano-and microMOF samples after degradation was modified (Fig. 5). Before degradation, the spectrum was well described by means of 3 quadrupolar components (Fig. 5), in agreement with the crystallographic MIL-100(Fe) structure, while after degradation, quadrupolar doublets with broadened lines were observed. The best physical description requires a discrete distribution of quadrupolar doublets suggesting a structure close to that of a typical amorphous structure. The Mössbauer spectra recorded at 77 K are similar to those obtained at 300 K at low velocity scale and their corresponding hyperfine structures confirm previous conclusions (Fig. S2). A deep analysis of the 12 mm/s spectra at 77 K allows a small magnetic component (estimated at about 2% in atomic Fe) to be observed, as displayed on the insets of Fig. S2, where relative transmission scale has been strongly extended. It corresponds clearly to hematite in the case of the degraded microMOFs while it could be attributed to poorly crystallized hematite (including traces of ultra-fine grains of goethite). Taking into account the presence of phosphates in the media surrounding the microMOFs, it is plausible that a highly disordered FeP based skeleton phase was formed, corroborating our previous hypothesis.

Energy dispersive X-ray analysis (EDX) was performed in order to obtain the Fe/P ratios of the intact MOFs, half degraded microMOFs, and totally degraded microMOFs. The results showed that there is a significant reduction of Fe/P ratios from 1000 (intact MOFs) to 7.6 (half degraded microMOFs) and 0.9 (totally degraded microMOFs), confirming that P was coordinated inside the porous MOF structures.

To demonstrate the progressive coordination of phosphates during the degradation process, Infra-Red (IR) spectroscopy was performed out on micro-MOFs at different degradation times (see Fig. S3). In addition to the decrease in intensity of the carboxylate stretching bands at ca. 1390 and 1580 cm^−1^, one could observe the progressive increase of intensity of the phosphate bands centered at 1050 cm^−1^. Although one cannot exclude that amorphous Fe oxide is also formed during the degradation process, this confirms the progressive incorporation of phosphates throughout of the particles when dispersed into PBS. The broadness of the phosphate bands is also in agreement with the amorphous character of the degradation product.

### MOFs stability during drug loading

Gem-MP has been successfully loaded in MIL-100(Fe) MOFs, following the previous methodology, which acted as efficient “nanosponges”, soaking the drugs from their aqueous solutions to reach within minutes unprecedented amounts (up to 30 wt%) of drug with loading efficiencies higher than 98%^24^. This is due to the coordination of the phosphate group of the drug on the Fe metal sites. In this study, the results from Gem-MP quantification by HPLC showed a high payload of 25 wt%. It is therefore important to investigate if drug loading induces a degradation of the MOF structure as it was the case when the MOFs were incubated in the presence of phosphates in PBS.

**Figure 6:**
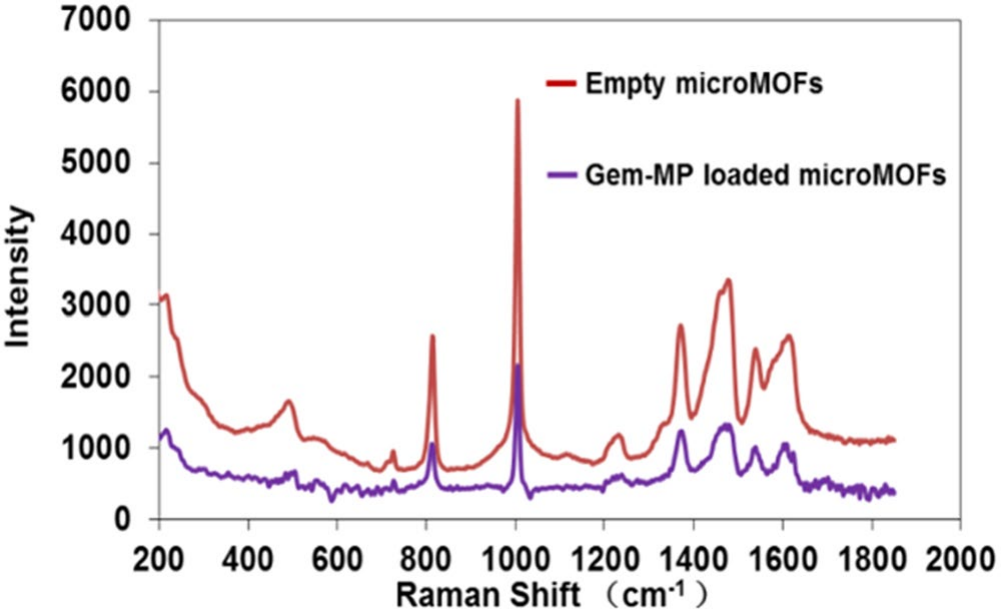
Effect of loading of Gem-MP in MIL-100(Fe) on the Raman spectra of the particles. MOFs particles before (red) and after (purple) Gem-MP loading.

The drug loaded particles were studied by Raman microscopy. Gem-MP did not present characteristic Raman peaks in the studied region, facilitating the studies. Figure 6 does not reveal any significant differences in the MIL-100(Fe) microMOFs Raman spectra before and after drug loading. These studies are in agreement with HPLC investigations, showing less than 2% of loss of the constitutive trimesate. Of note, the microMOFs maintained their crystalline structure after Gem-MP loading^24^. In a nutshell, the MOF 3D structure was maintained after drug loading with no impact of the drug on the MOFs long-range or local structure.

Previously we showed that MOFs immersed in PBS degraded with kinetics dependent upon the phosphates concentration. When Gem-MP was loaded in MOFs at the maximum payload of 30 wt%, the equivalent amount of phosphates was 0.038 mM, whereas the molar ratio P/Fe was only 0.38, which could explain why MOFs did not degrade during drug entrapment.

### MOF stability during coating

**Figure 7:**
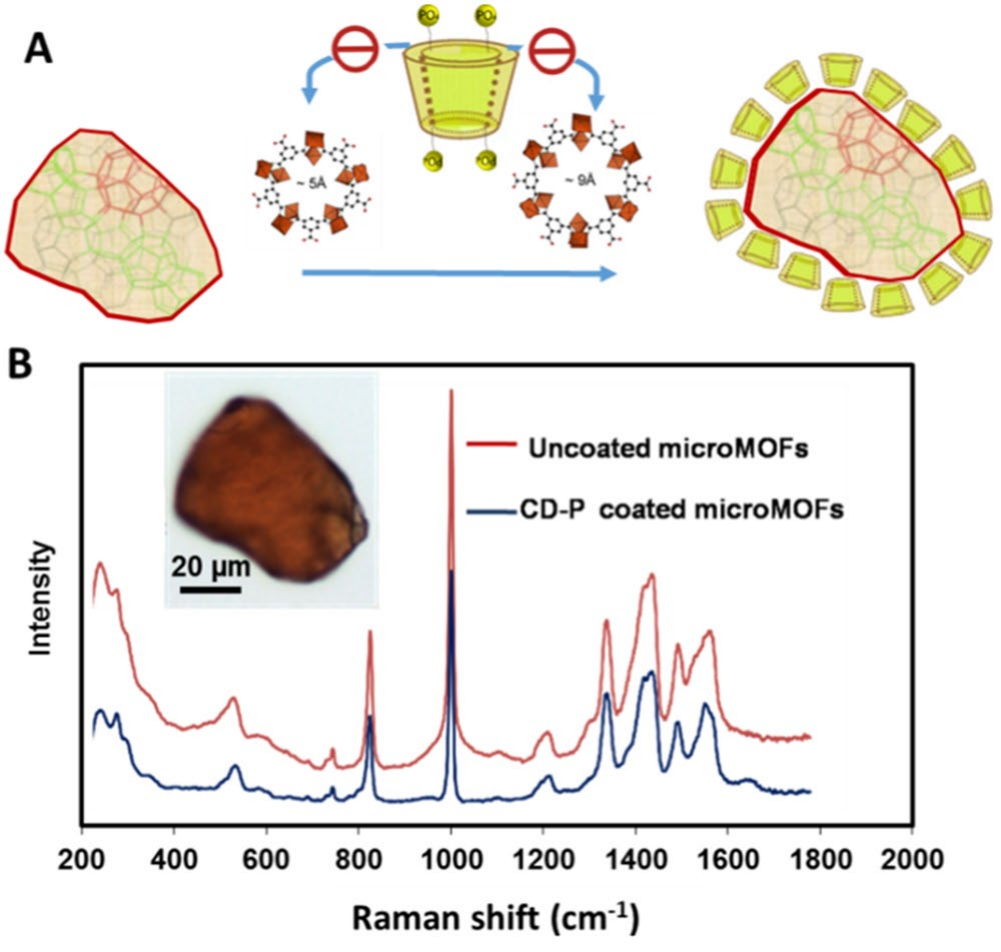
Raman spectra of individual MIL-100(Fe) MOF particles, coated or not with P-CD. Schematic representation of the coating procedure (**A**) and experimental results (**B**) for MOF particles before and after CD-P coating.

Cyclodextrins (CDs) bearing several phosphate anchoring groups (CD-P) were successfully used to functionalize the surface of MOF particles^33^. Taking advantage of the fact that the size of CD-P is larger than the MIL-100(Fe) windows (Fig. 7) the coating could be achieved by a simple way consisting in incubating the MOFs with CD-P in water. This “green” method allowed the formation of CD-P shells within minutes and the coating was stable, even in biological media^33^, due to the cooperative coordination between phosphates on CD-P and Fe sites at the MOF’s surface.

In this study, CD-P coatings were produced on microMOFs following the previous protocol^33^. As reported in the previous work^33^, elemental analysis was used to evaluate the coating efficacy of grafting CD-P, showing that around 17 wt% of CD-P was associated to the MOFs. The particles, before and after coating, were studied by Raman microscopy. Of note, CD-P did not present any specific peak in the observed region. It was found that after coating, the particles maintained same morphology, color and Raman spectra, indicating that CD-P surface modification did not induce particle degradation. Similarly as in the case of drug loading, the P/Fe ratio in CD-P coated microMOFs was lower than 1 (only 0.6), which could explain why the microMOFs did not degrade after coating. The good stability of the particles after CD coating offer thus promising possibilities to load drugs of interest and graft ligands for targeting purposes.

## Conclusion

Whatever their size, MIL-100(Fe) MOFs degrade in PBS losing their constitutive trimesate linkers and eventually became amorphous with a clear impact of the phosphate concentration and the particle size: the higher the phosphate concentration and the lower the particle the faster the degradation of the MOF particles. Interestingly, nano-and microMOFs both kept their initial sizes during degradation but transformed into inorganic edifices. Raman microscopy enabled gaining insights on the degradation mechanism and showed that intact cores were separated from the eroded regions by clearly visible erosion fronts. Iron phosphates were identified by Mössbauer spectrometry (and IR spectroscopy) as main components in the eroded regions. In contrast, neither drug entrapment nor particle coating did affect the integrity of the tridimensional MOF network due to the more diluted conditions. These findings are of interest in the development of stable drug-loaded MOF particles. Further studies will be focused on the degradation of other engineered MOF materials of interest in biomedicine.

## Materials and Methods

### Chemicals

Iron (III) chloride hexahydrate (98%, Alfa Aesar, Schiltigheim, France), 1,3,5-benzenetricarboxylic acid (BTC, 95%, Sigma-Aldrich, Saint-Quentin-Fallavier, France) and absolute ethanol (99%, Carlo Erba, Val-de-Reuil, France) were used for MOF synthesis. PBS (11.9 mM, pH 7.4 ± 0.1) was purchased from Life Technology Co., Ltd. (Saint Aubin, France). It contains 11.9 mM phosphates, 137 mM NaCl and 2.7 mM KCl. Gem-MP (CH_12_F_2_N_3_OP) was purchased from Toronto Research Chemicals, Canada. Phosphated β-cyclodextrin sodium salt (CD-P, Cyclolab, CY-2017.1, molecular formula: C_42_H_70_O_47_P_4_Na_4_) was used for coating. Milli-Q water was obtained from a Millipore apparatus equipped with a 0.22 μm filter. Reagents and solvents were used without further purification.

### Nano-and micro-MOFs synthesis

MIL-100(Fe) iron trimesate nanoMOFs were synthesized by adapting a previously described method^1^. Briefly, nanoMOFs were obtained by microwave-assisted hydrothermal synthesis from a mixture of iron chloride (8.97 mmol) and BTC (4.02 mmol) in 20 mL of deionized water. The mixture was heated for 6 min at 130 °C under stirring. The power applied was 800 Watts (Mars-5, CEM, US) with a power maximum output 1,600 ± 240 Watts and frequency at full power 2,450 mHz. The resulting nanoMOFs, recovered by centrifugation, were washed with absolute ethanol 6 times to remove the residual non reacted organic acid. A last centrifugation at 5500 × g was performed during 1 min in absolute ethanol to sediment the largest particles and recover the supernatants as a suspension of monodisperse nanoparticles. Nanoparticles were stored wet in ethanol until final use.

To prepare microMOFs, a reaction mixture composed of 2.7 g iron (III) chloride hexahydrate (10.00 mmol) and 2.1 g trimesic acid (10.00 mmol) in 50 mL of water, was placed in a large Teflon bomb under magnetic stirring for 15 minutes. The Teflon reactor was encased in a metal bomb with controlled pressure, before being placed in an autoclave with a 1 hour heating ramp to 130 °C, and held at this temperature for 72 h. The product was then cooled before being filtered and was then washed by heating under reflux, first in 700 mL of ethanol at 75 °C for 2 h and finally in 700 mL of water at 90 °C for 2 h. The product was then collected by filtration under vacuum and stored as powder after being dried in air.

### MOFs characterization

The crystallinity and purity of MIL-100(Fe) particles were assessed by PXRD patterns which were collected in a conventional high resolution (θ–2θ) D5000 Bruker diffractometer (λ_Cu_ K_α_, K_α2_) from 1° to 20° (2θ) using a step size of 0.02° and 4° per step in continuous mode. The morphologies of microMOFs were investigated by Raman microscopy (Malvern^®^ Morphologi G3-ID). Briefly, optical images of more than 2000 particles were captured and statistical analysis of size and circularity were carried out.

Dynamic light scattering (DLS) and Nanoparticle tracking analysis (NTA) were performed by a Zetasizer (Malvern^®^ Nano-ZS90) and Nanosight (Malvern^®^ LM10), to characterize the size distribution of nanoMOFs. Zeta potential of nanoMOFs was characterized by a Zetasizer (Malvern^®^ Nano-ZS90). Nanosight analysis is a combination of a conventional optical microscope and a laser to illuminate the nanoparticles introduced into the visualizing unit with a 1.0 mL syringe system. Individual particles could be visualized as point-scatter moving under Brownian motion. Measurements were performed for 5 times over a period of 60 s at 25 °C. The particle size and concentration were analyzed using the NTA software. Transmission electron microscopy (TEM) images for nanoMOFs were collected using a JEOL^®^ JEM-2200FS microscope. The circularity of nanoMOFs was obtained from TEM images and analyzed by Origin 9.0 software.

### Statistical analysis of MIL-100(Fe) degradation

The size distribution and morphology of MOFs after degradation in PBS were characterized. Briefly, Raman microscopy was applied for microMOFs to investigate their size and circularity before and after incubation in PBS (11.9 mM) for 8 days. TEM was used for nanoMOFs to observe the morphological variation after incubation in PBS (1.19 mM) for 6 h. DLS was used to characterize the mean diameter variation during degradation of nanoMOFs in PBS with different concentrations (11.9 mM, 1.19 mM and 0.12 mM).

### HPLC determination of the Gem-MP loading and trimesate release

The detection of Gem-MP and trimesate was carried out in HPLC (Agilent 1100, USA) connected to a Phenomenex C18 column (4.6 × 250 mm, 5 µm) at a flow rate of 1.0 mL/min. For the analysis of Gem-MP, a mobile phase consisting of 84% buffer (0.2 M TEAA):16% MeOH was used. It was detected at 254 nm with an injection volume of 20 µl. Analysis of trimesate was performed with a mobile phase containing 90% buffer (5.75 g/L of NH_4_H_2_PO_4_): 10% Acetonitrile (5 mM TBAP). The injection volume was 5 µl and the detection wavelength was set at 220 nm.

### Individual analysis of MIL-100(Fe) degradation

Individual particles were observed by Raman microscopy and Raman spectra were obtained for degraded and non-degraded particles. Microparticles were kept in PBS (11.9 mM) for 8 days under the monitor of Raman microscopy. Images were captured every 30 min to track the degradation process using automated imaging. Surface areas and intensity mean were extracted from the software and kinetic analysis was carried out in function of surface areas and intensity mean. Raman spectra were recorded at the laser wavelength of 785 nm. The laser spot was 3 μm in diameter with power output as 15 mW.

Transmission ^57^Fe Mössbauer spectrometry was performed using a conventional electromagnetic transducer with a constant acceleration and a ^57^Co/Rh γ-ray source. Mössbauer spectra were recorded at 300 K and 77 K using a bath cryostat on samples which consist in a powdered layer containing about 5 mg/cm^2^ of Fe/cm^2^. The hyperfine structure was modeled by a least-square fitting procedure involving quadrupolar doublets and/or Zeeman sextets composed of lorentzian lines using unpublished program ‘MOSFIT’. The isomer shift values (IS) are referred to that of α-Fe at 300 K, used as a standard to calibrate the spectrometer.

### EDX analysis

The chemical compositions of the samples were determined by EDX performed on a FEI Magellan 400 Scanning Electron Microscope. Three samples were analyzed: i) intact MOFs; ii) half degraded microMOFs; iii) totally degraded microMOFs. Twelve spectra were obtained for each sample and the content of P, Fe, and Cl was calculated by getting the average of the 12 spectra. Finally, Fe/P ratios were calculated for each sample.

### PXRD analysis

PXRD patterns were collected in reflection mode using a PANalytical Empyrean Series 2 diffractometer with an average wavelength of 1.541 Å Cu Kα (45 kV and 40 mA). Measurements were performed at room temperature between 3° and 100° in 2θ, using a step size of 0.026° step, and a counting time per step of 200 s.

### Investigation of drug loaded MIL-100(Fe)

Gem-MP was loaded in microMOFs by adapting a previously described method^24^. Briefly, MOFs in ethanol were re-dispersed in water under gentle magnetic stirring overnight. Then Gem-MP loaded MOFs were prepared by soaking for one week microMOFs in Gem-MP aqueous solution (0.5 mg/mL) using a weight ratio 1:2 (Gem-MP: microMOFs) to reach maximal loadings of 30 wt%. Afterwards, Raman spectra of Gem-MP loaded MOFs were obtained by Raman microscopy.

### Investigation of CD-P coated MIL-100(Fe)

MIL-100(Fe) MOFs were coated with CD-P as previously described^33^. Briefly, MOFs were suspended in a CD-P aqueous solution under gentle stirring at room temperature for 24 h. The initial MOFs/CD-P weight ratio was 1:0.5, and the final MOFs concentration was 4 mg/mL. The coated MOFs were recovered by centrifugation (5000 g for 5 min) and were washed with deionized water in order to remove the excess CD-P which was not associated to the MOFs surface. Finally, Raman spectra and images of CD-P coated MOFs were recorded by Raman microscopy.

## Electronic supplementary material

Supplementary information 
