# Supplementary material for: New insights into the degradation mechanism of metal-organic frameworks drug carriers
Source: Sci Rep. 2017 Oct 13;7:13142. doi: 10.1038/s41598-017-13323-1 (PMC5640595; doi:10.1038/s41598-017-13323-1)
Supplement: Supplementary file 1 — Supplementary information [file 41598_2017_13323_MOESM1_ESM.pdf]

Supplementary Information

New insights into the degradation mechanism of metal-organic frameworks drug carriers

X. Li[1], L. Lachmanski[2], S. Safi[2], S. Sene[3], C. Serre[3], J.M. Greneche[4], J. Zhang[5], R. Gref[1*]

[1] *Institut des Sciences Moléculaires d'Orsay, UMR 8214 CNRS, Université Paris-Sud, Université Paris-Saclay, Orsay, 91405, France*

[2] *Malvern Instruments, 30 rue Jean Rostand, Orsay, 91405, France*

[3] *Institut de Matériaux Poreux de Paris, FRE 2000 CNRS ENS-ESPCI, PSL Research University, Paris, 75005, France*

[4] *Institut des Molécules et des Matériaux du Mans (IMMM) - UMR 6283 CNRS, Le Mans, 72085 France*

[5] *Shanghai Institute of Materia Medica, Chinese Academy of Sciences, Shanghai, 201203, China*

*Corresponding author: E-mail address: ruxandra.gref@u-psud.fr

Supplementary Methods

Trimesate release from nanoMOFs in PBS. NanoMOFs were incubated in PBS (11.9 mM, 1.19 mM and 0.12 mM) at 37°C. The samples were centifugated and the amount of trimesate in the supernatant was assessed by HPLC. Analysis of trimesate was performed with a mobile phase containing 90% buffer (5.75 g/L of $NH_4H_2PO_4$): 10% Acetonitrile (5 mM TBAP). The injection volume was 5 µl and the detection wavelength was set at 220 nm. As controls, MOFs were incubated in water instead of PBS under the same conditions and trimesate release was monitored.

PXRD analysis. The crystallinity and purity of MIL-100 (Fe) nanoMOFs before and after degradation were assessed by PXRD. PXRD patterns were collected using a high resolution (θ-2θ) D5000 Bruker diffractometer (λ_{Cu} K_α, $K_{\alpha 2}$) from 1° to 90° (2θ) using a step size of 0.02° and 4° per step in continuous mode. Four samples were used for the analysis: i) intact nanoMOFs; ii) totally degraded nanoMOFs after incubation in PBS (11.9 mM) for 4 h; iii) half-degraded microMOFs after incubation in PBS (11.9 mM) for 3 days, and iv) totally degraded microMOFs after incubation in PBS (11.9 mM) for 1 month.

Fourier transform infrared spectroscopy (FTIR) analysis. 100 mg of microMOFs were incubated in 50 mL of PBS (11.9 mM) at 37 °C with stirring speed at 100 rpm. The samples were collected by centrifugation after incubation for 1 h, 2 h, 4 h, 6 h, 12 h, 24 h, 3 d, 5 d, and 7 d. The samples were dried at 100°C overnight. IR spectra were recorded in KBr disk on a Nicolet 205 FTIR.

Supplementary Results

NanoMOFs degraded quickly in concentrated PBS (11.9 mM), with their crystallinity lost (Fig. S1) and 32 ± 3.0 wt % of trimesate released after 4 hours incubation (Table S1). When PBS was diluted to 1.19 mM, it took 6 hours to get totally degraded, releasing 34 ± 2.6 wt % of trimesate. NanoMOF can also be degraded even in extremely diluted PBS (0.12 mM), releasing 31 ± 2.8 wt % after two days incubation. Whereas, there was no degradation observed in water after 2 days incubation, with less than 2 wt % of their trimesate released and the crystallinity well preserved.

Table S1. Trimesate release of nanoMOF in PBS (11.9 mM, 1.19 mM, and 0.12 mM) and in water during incubation at 37°C.

PBS concentration (mM)	Incubation time (h)	Trimesate release (wt%)
11.9	4	32 ± 3.0
1.19	6	34 ± 2.6
0.12	48	31 ± 2.8
0 (water)	48	≤ 2 ± 0.2

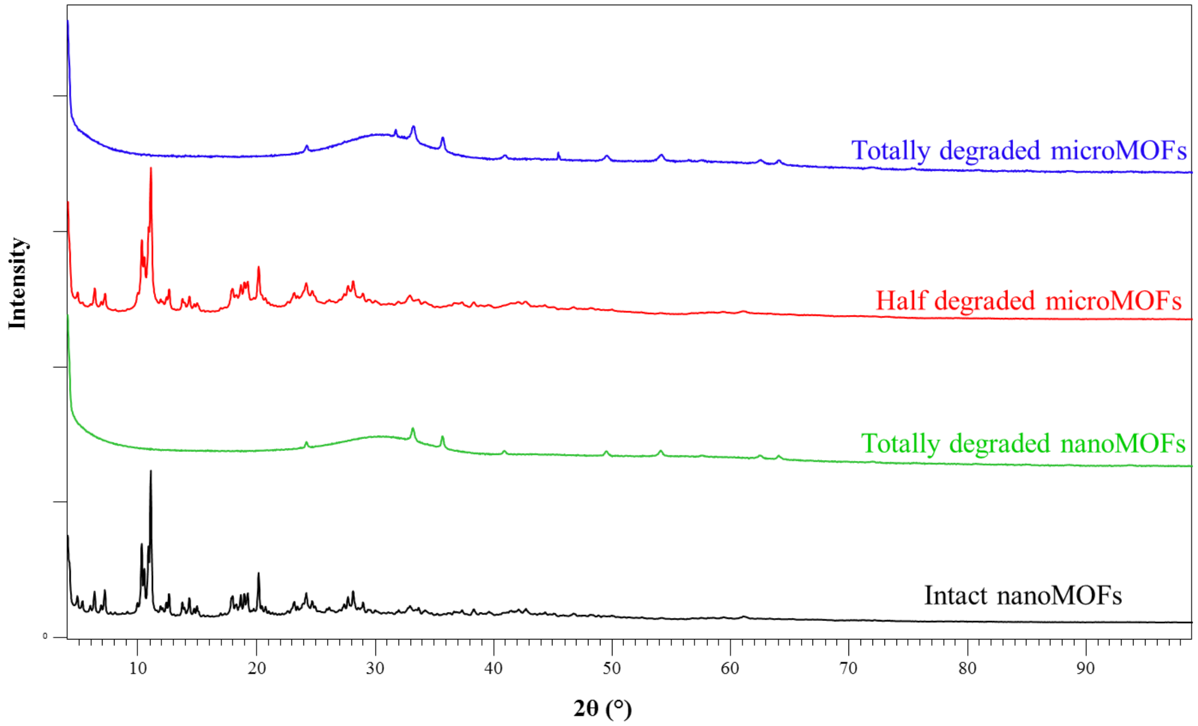

Fig. S1. PXRD pattern of intact nanoMOFs (black), totally degraded nanoMOFs (green), half-degraded microMOFs (red), and totally degraded microMOFs (blue).

The Mössbauer spectra at 77K of degraded nanoMOF and microMOF are illustrated in Fig. S2. Experiments were performed at 2 and 12 mm/s. The low velocity scale spectra are similar to those obtained at 300K and their corresponding hyperfine structures confirm previous conclusions. A deep analysis of the 12 mm/s spectra at 77K allows a small magnetic component (estimated at about 2% in at Fe) to be observed, as displayed on the insets of Fig.S2, where relative transmission scale has been strongly extended. It corresponds clearly to hematite in the case of the degraded microMOFs while it could be attributed to poorly crystallized hematite (including traces of ultra-fine grains of goethite).

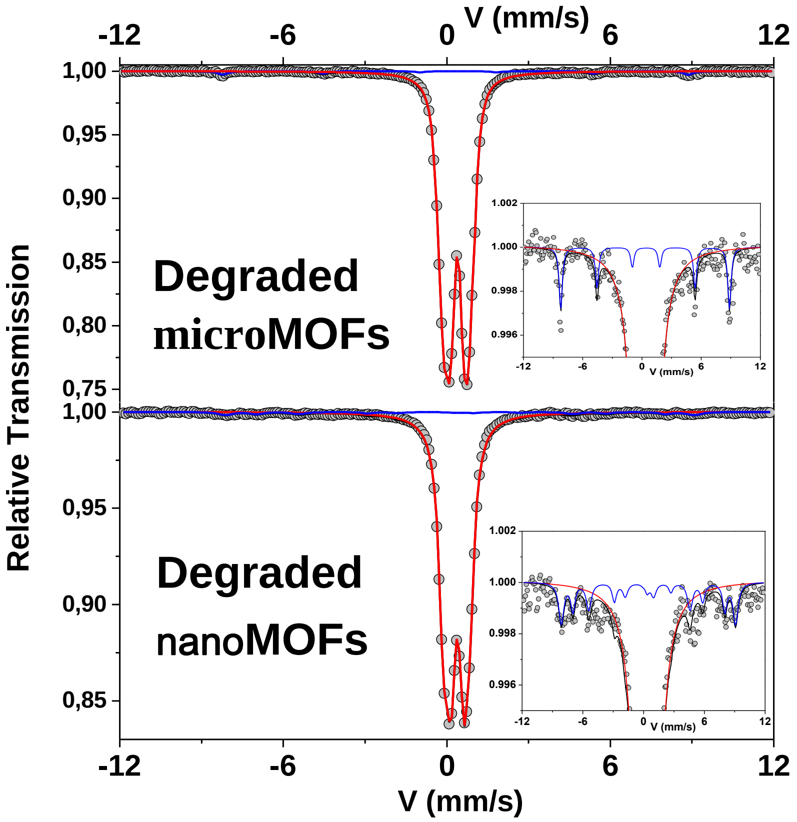

Fig. S2 Mössbauer spectra at 77K of degraded nanoMOF and microMOF

Energy dispersive X-ray analysis (EDX) was performed in order to obtain the Fe/P ratios of the intact MOFs, half degraded microMOFs, and totally degraded microMOFs. The results showed that there is significant reduce of Fe/P ratios from 1000 (intact MOFs) to 7.6 (half degraded microMOFs) and 0.9 (totally degraded microMOFs), confirming that P was coordinated inside the amorphous matrices of MOF structure during the degradation process.

Table S2. Fe/P ratios of the intact MOFs, half degraded microMOFs, and totally degraded microMOFs.

Type of MOFs	Fe (%)	P	Fe/P
intact	92.55 ± 1.43	0.09 ± 0.41	~ 1000
Half-degraded	74.94 ± 3.10	9.89 ± 3.64	~ 7.6
Totally degraded	42.60 ± 11.98	44.57 ± 13.26	~ 0.9

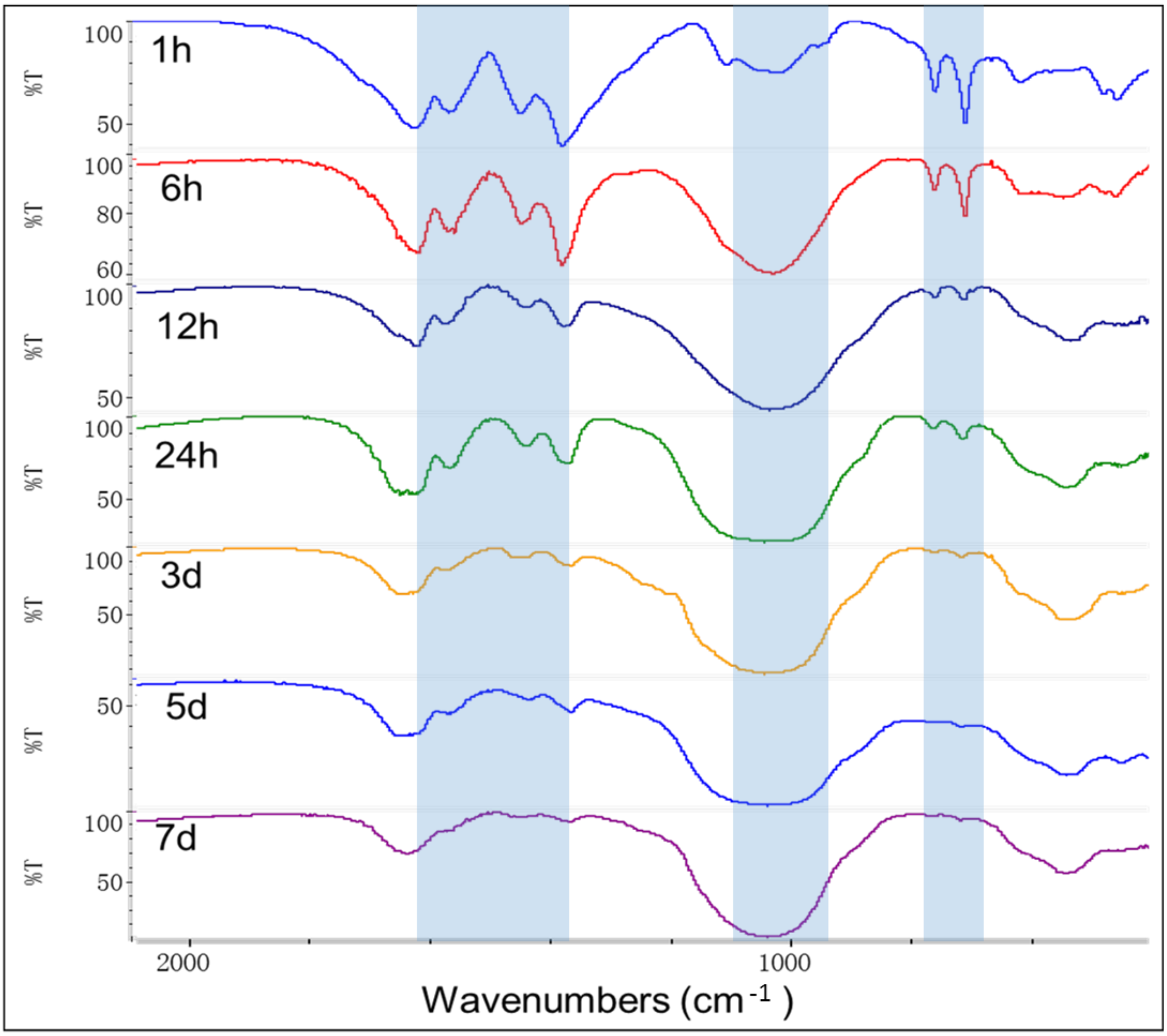

Fig. S3 FTIR spectra of microMOFs during their degradation in PBS
